# Upper gastrointestinal symptoms in autoimmune gastritis: A cross-sectional study

**DOI:** 10.1097/MD.0000000000005784

**Published:** 2017-01-10

**Authors:** Marilia Carabotti, Edith Lahner, Gianluca Esposito, Maria Carlotta Sacchi, Carola Severi, Bruno Annibale

**Affiliations:** a Department of Internal Medicine and Medical Specialties, University Sapienza, viale del Policlinico 155, 00161, Rome, Italy; b Department of Medical and Surgical Sciences and Traslational Medicine, University Sapienza, Via di Grottarossa 1035, 00189, Rome, Italy.

**Keywords:** autoimmune gastritis, dyspepsia, gastrointestinal symptoms, pernicious anemia

## Abstract

Autoimmune gastritis is often suspected for its hematologic findings, and rarely the diagnosis is made for the presence of gastrointestinal symptoms. Aims of this cross-sectional study were to assess in a large cohort of patients affected by autoimmune gastritis the occurrence and the pattern of gastrointestinal symptoms and to evaluate whether symptomatic patients are characterized by specific clinical features.

Gastrointestinal symptoms of 379 consecutive autoimmune gastritis patients were systematically assessed and classified following Rome III Criteria. Association between symptoms and anemia pattern, positivity to gastric autoantibodies, *Helicobacter pylori* infection, and concomitant autoimmune disease were evaluated.

In total, 70.2% of patients were female, median age 55 years (range 17–83). Pernicious anemia (53.6%), iron deficiency anemia (34.8%), gastric autoantibodies (68.8%), and autoimmune disorders (41.7%) were present. However, 56.7% of patients complained of gastrointestinal symptoms, 69.8% of them had exclusively upper symptoms, 15.8% only lower and 14.4% concomitant upper and lower symptoms. Dyspepsia, subtype postprandial distress syndrome was the most represented, being present in 60.2% of symptomatic patients. Univariate and multivariate analyses showed that age <55 years (OR 1.6 [CI:1–2.5]), absence of smoking habit (OR 2.2 [CI:1.2–4]), and absence of anemia (OR 3.1 [CI:1.5–6.4]) were independent factors associated to dyspepsia.

Autoimmune gastritis is associated in almost 60% of cases with gastrointestinal symptoms, in particular dyspepsia. Dyspepsia is strictly related to younger age, no smoking, and absence of anemia.

## 1 Introduction

Autoimmune gastritis (AG) is a chronic disease occurring in up to 8% of the general population.^[1]^ This condition is characterized by loss of the oxyntic glands with consequent hypochlorhydria, lack of intrinsic factor production, and, in a later stage, pernicious anemia. Often, the positivity of autoantibodies against parietal cells and/or intrinsic factor, the co-presence of autoimmune diseases as thyroid autoimmune disease or type 1 diabetes are associated.^[2–5]^ Histologically AG is characterized by gastric body atrophy, defined as replacement of oxyntic glands by metaplastic pyloric or intestinal glands according to the updated Sydney System.^[6]^

A frequent clinical presentation of AG is pernicious anemia, a megaloblastic anemia arising from vitamin B_12_ malabsorption as a consequence of intrinsic factor deficiency,^[7,8]^ or the iron deficiency anemia due to iron malabsorption as a consequence of reduced gastric acid secretion together with normal or low cobalamin levels^[9]^ and some of these patients may over time develop overt pernicious anemia.^[10]^ Less frequently, the suspicion of AG arise from the presence of gastrointestinal (GI) symptoms. From the GI point of view, the clinical spectrum of AG has been traditionally considered almost silent, even if the occurrence of symptoms in this population has been reported, but risk factors for GI symptoms associations in AG have not be established.^[11–13]^

The aim of this study was to assess in a large cohort of AG patients the occurrence and pattern of GI symptoms and to evaluate whether AG patients with GI symptoms are characterized by specific clinical features.

## 2 Methods

### 2.1 Patients and study design

A cohort of 379 consecutive AG patients, diagnosed from 1995 to 2013 in a tertiary referral center, was analyzed. Gastroscopy was performed in 78.9% (299/379) of cases for pernicious or iron deficiency anemia, in 8.4% (32/379) for GI symptoms, in 2.9% (11/379) for neurologic or endocrine disorders, in 2.9% (11/379) for increased levels of gastrin, and 6.9% (26/379) for other causes. Eventually proton pump inhibitors treatment was withdrawn at least 2 weeks before gastroscopy.

The presence and the frequency of GI symptoms (heartburn, regurgitation, early satiety, nausea, vomiting, postprandial fullness, epigastric pain, abdominal pain, constipation, diarrhea, and bloating) were assessed at the time of the first visit through a standardized interview, according to a form that we have been using for many years in our GI Department. Upper GI symptoms were analyzed either separately or altogether to classify patients in defined syndromes which may overlap. Gastro-esophageal reflux disease (GERD) was defined by the presence of typical reflux symptoms such as troublesome heartburn and/or regurgitation at least once-2 times a week.^[14]^ Dyspepsia was defined by Criteria of Rome III for functional disorders, by the presence of symptoms thought to originate in the gastroduodenal region, with symptoms onset at least 6 months before.^[15]^ This was further subdivided in the postprandial distress syndrome (PDS) in the presence of bothersome post-prandial fullness and/or early satiation at least several times per week and epigastric pain syndrome (EPS) in the presence of pain or burning localized in the epigastrium and not associated with other abdominal or chest-related symptoms, at least once per week for the last 3 months. Since the study dealt with patients affected by AG, PDS and EPS dyspepsia will be indicated as PDS- and EPS-like throughout this article. In the presence of lower GI symptoms, patients were categorized as having the irritable bowel syndrome (IBS), further subdivided into diarrhea-predominant IBS (IBS-D), constipation-predominant IBS (IBS-C), or mixed stool pattern IBS (IBS-M), functional constipation, functional diarrhea, and functional bloating following Rome III Criteria.^[16]^ In the presence of abdominal pain and without symptoms to meet criteria for other functional GI disorder that could explain the pain, the diagnosis of functional abdominal pain syndrome, following Rome Criteria was made.^[17]^ Informed consent was obtained from each participant. For this cross-sectional study, an ethical approval was not requested.

### 2.2 Diagnosis of autoimmune gastritis, *Helicobacter pylori,* and autoimmune status

AG has been defined on the basis of the concomitant presence of fasting hypergastrinaemia, low pepsinogen I level, histological confirmation of body atrophy, positivity to antiparietal cells, and/or intrinsic factor antibodies and/or compresence of other autoimmune diseases as previously reported.^[18]^ The anemia pattern was assessed by evaluating hemoglobin, mean corpuscular volume (MCV), ferritin, and vitamin B_12_ values. Pernicious anemia was defined as the low hemoglobin concentration, MCV>100 fl together with low B_12_ vitamin levels, responding to intramuscular B_12_ vitamin treatment. Iron deficiency anemia was defined as the low hemoglobin concentration, MCV< 80 fl, and ferritin<30 ng/mL.^[19,20]^ Antiparietal cells’ antibodies were evaluated on serum using a solid-phase immunosorbent assay commercial kit (Autostat, Cogent Diagnostic Ltd, Edinburgh, UK)^[21,22]^ and antibodies against intrinsic factor were assessed using a commercial kit (Quanta Lite, INOVA Diagnostics, San Diego, CA).^[19]^ Diagnosis of *H pylori* infection was assessed on the basis of the bacteria evident at histological examination (Giemsa stain) together with infiltration of polymorphonuclear cells in gastric mucosa.^[23]^ The diagnosis of autoimmune thyroid and extrathyroid disease was assessed as previously described.^[5]^

### 2.3 Statistical analysis

Data were expressed as percentage (%) of total, or median (range). Univariate analyses were performed by Mann–Whitney and Fisher's exact tests for categorical and continuous variables in order to identify clinical differences between symptomless and symptomatic patients. Odds ratio (OR) and 95% confidence intervals (CIs) were used to describe associations and were obtained by logistic regression analysis. Two-tailed *P* values <0.05 were considered statistically significant. The statistical analysis was carried out using a dedicated software package (MedCalc Software, Mariakerke, Belgium, version 12.2).

## 3 Results

Of the 379 patients included, 70.2% (n = 266) were female, with a median age of 55 years (17–83 years). Smoking habit was present in 19% (n = 72) of patients. Clinical features associated to AG were: pernicious anemia (53.6%), iron deficiency anemia (34.8%), presence of gastric autoantibodies (anti-parietal cell and/or anti-intrinsic factor) (67.3%), autoimmune disorders (41.7%), and *H pylori* infection (26.1%). Concerning autoimmune disorders, 89% of AG patients had thyroid disorders and 11% had 1 or 2 associated extra-thyroid diseases (i.e., vitiligo, alopecia, diabetes, rheumatoid arthritis, hemolytic anemia, Sjögren's syndrome, psoriasis, autoimmune hepatitis, and myasthenia). None of the patients had GI cancer at the time of AG diagnosis. Polypharmacy (use of more than 2 drugs) was present in 5.5% (n = 21) of patients for common conditions such as cardiovascular diseases, dyslipidemia, diabetes, and osteoporosis. Prokinetics were taken by 6.6% (n = 25) of patients.

### 3.1 Occurrence of GI symptoms

One or more GI symptoms were present in 56.7% (n = 215) of AG patients. Table 1 shows the clinical features of AG patients with respect to the presence or the absence of GI symptoms.

**Table 1:**
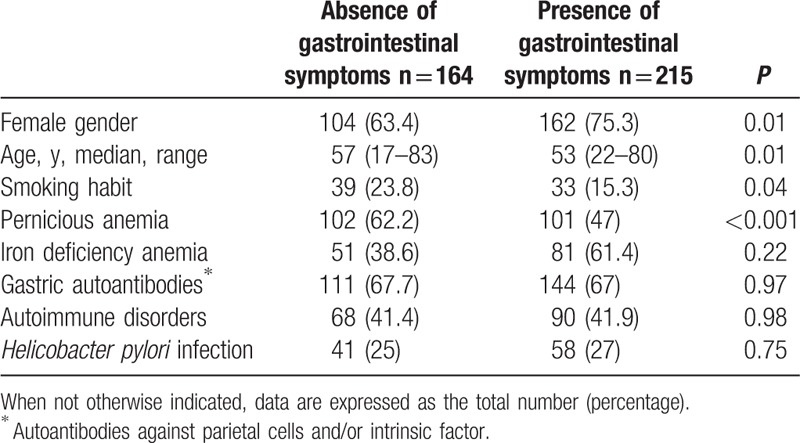
Clinical features of patients with autoimmune gastritis with respect to the presence or the absence of gastrointestinal symptoms.

**Figure 1:**
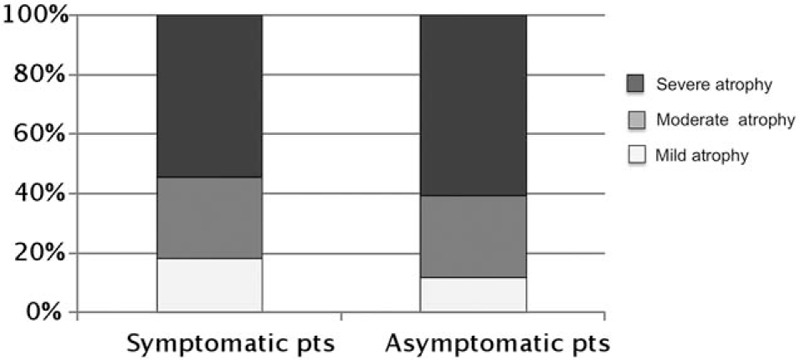
Severity degree of gastric atrophy in symptomatic and asymptomatic autoimmune gastritis patients. pts = patients.

Females were significantly prevalent, about 10% more, among the patients with GI symptoms compared to those without. Symptomatic patients were significantly younger, about 5 years, and more often no smokers, about a third less, in comparison to the asymptomatic ones. Pernicious anemia was significantly more frequent, about a third more, in the asymptomatic group in comparison to the symptomatic group. No differences were found between symptomatic and asymptomatic patients concerning the presence of iron deficiency anemia, positivity to gastric autoantibodies, prevalence of *H pylori* infection, and the concomitant presence of autoimmune disorders. The Sydney score of corporal atrophy was evaluated in patients with and without GI symptoms. As shown in Fig. 1, the distribution of mild, moderate, and severe corporal atrophy was not significantly different between the 2 groups of AG patients.

### 3.2 GI symptomatic pattern

**Figure 2:**
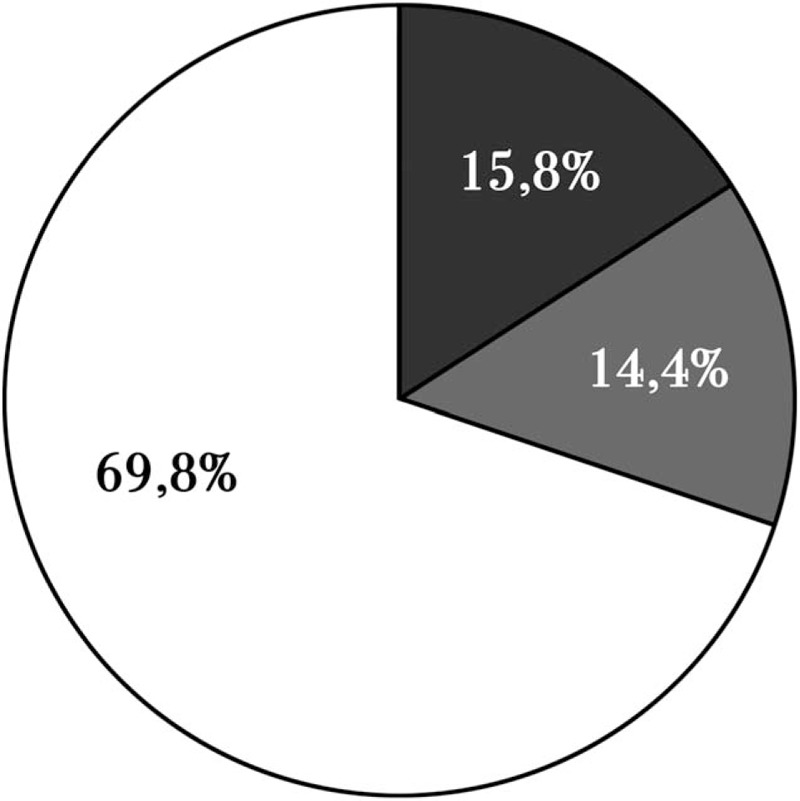
Symptomatic pattern of autoimmune gastritis patients. Black = only lower gastrointestinal symptoms, Gray = combined of upper and lower gastrointestinal symptoms, White = only upper gastrointestinal symptoms.

Figure 2 illustrates the GI symptoms complained by AG patients. Among symptomatic patients, the large part had exclusively upper GI symptoms (n = 150) and a smaller part complained respectively only lower GI symptoms (n = 34) or concomitant upper and lower GI symptoms (n = 31).

Analyzing all patients with upper GI symptoms (n = 181), 60.2% (n = 109) reported PDS-like dyspepsia, 3.8% (n = 7) EPS-like dyspepsia, 7.2% (n = 13) overlap PDS and EPS-like dyspepsia, 7.2% (n = 13) GERD, 17.7% (n = 32) GERD and dyspepsia, and 3.8% (n = 7) nausea and/or vomiting. Among all patients with lower GI symptoms (n = 65), 27.7% (n = 18) reported the functional abdominal pain syndrome, 3.1% (n = 2) IBS-C, 3.1% (n = 2) IBS-D, 16.9% (n = 11) IBS-M, 21.5% (n = 14) functional constipation, 12.3% (n = 8) functional diarrhea, and 15.4% (n = 10) functional bloating.

Logistic regression analysis was performed in order to assess the presence of clinical features associated to the presence of upper GI symptoms in AG patients, which resulted to be more frequent in the study population (Table 2). This analysis showed that the younger age (<55 years), the absence of smoking habit, and the absence of anemia were independent factors associated to the presence upper GI symptoms in AG patients. The positive association between the absence of anemia and the presence of GI symptoms was confirmed when the absence of pernicious anemia (OR 2.4 [95%CI: 1.2–5.2]) and iron deficiency anemia (OR 3.1 [95%CI: 1.4–6.8]) was separately considered. When the presence of PDS-like dyspepsia in AG patients was considered as the dependent variable, the positive association with the same independent variables was shown (Table 2).

**Table 2:**
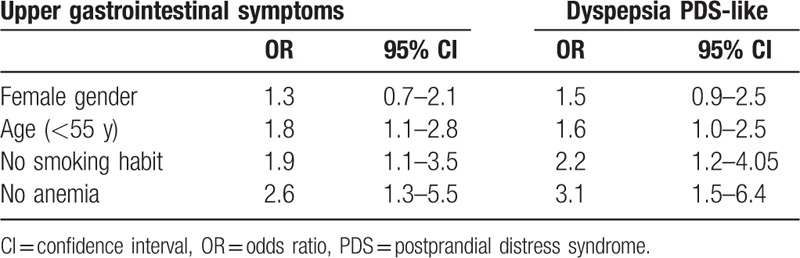
Clinical predictive factors associated to upper gastrointestinal symptoms in patients with autoimmune gastritis.

## 4 Discussion

This study aimed to investigate, in a large cohort of AG patients, the occurrence and the pattern of GI symptoms and the presence of specific clinical features that could represent a risk factor for symptoms’ association. We demonstrated that more than half of AG patients have 1 or more GI symptoms. The major part (about 70%) of symptomatic AG patients complained of upper GI symptoms, in particular PDS-like dyspepsia characterized by post-prandial fullness and/or early satiation, which were the most frequent symptoms reported. Among the upper GI symptoms reported, a quarter was represented by GERD, alone or associated to dyspepsia. Despite the impaired acid secretion occurring in AG due to atrophy of the oxyntic mucosa, it is possible that nonacidic refluxes might develop and play as determinants of heartburn perception, as recently observed.^[24]^ In fact, in a recent paper, Tenca et al^[13]^ demonstrated that 24% of AG patients present gastroesophageal reflux at pH-impedence monitoring, irrespective of the presence of symptoms, confirming that reflux can occur in AG patients. In another study by Miceli et al,^[11]^ it has been reported that 24% of AG patients complained of heartburn and 12% of acid regurgitation. In addition, we recently describe a case of Barrett's esophagus in a patient affected by pernicious anemia, supporting that GERD and its related complications may originate even in a hypochlorhydric patient.^[25]^

In our population, about 16% and 14% of the symptomatic AG patients complained only of lower GI symptoms or of concomitant upper and lower GI symptoms, respectively. The proportion of patients who had concomitant upper and lower GI symptoms was lower among our AG patients than that reported in patients with functional dyspepsia in which the overlapping has been shown to be about 30%.^[26,27]^ This data might support the hypothesis that dyspepsia associated to AG is quite different from functional dyspepsia.

It is known that dyspepsia is one of the most prevalent upper GI disorders in the general population, and its prevalence is reported to be about 20%.^[15]^ In our cohort of AG patients, the prevalence of dyspepsia is about 2 times higher than that expected in the general population, suggesting a potential role of this condition in the arising of this symptom. A former study conducted by Miceli et al^[11]^ reported in 99 patients with AG, frequencies of postprandial fullness, and early satiety of 7.1% and 10.1%, respectively.^[11]^ These figures seem much lower compared to those observed in our study. In the study by Soykan et al,^[12]^ 46.7% of AG patients present abdominal symptoms, mainly characterized by abdominal bloating and pain, with a minor part complained of upper symptoms such as nausea and belching. It is possible that discrepancy between our results and the previously studies is mainly related to the different method used to collect symptoms.

Although the roles of many pathophysiological factors have been proposed in dyspepsia (i.e., altered gastro-duodenal motility, visceral hypersensitivity, *H pylori* infection, acid secretion, genetic, psychosocial, and lifestyle factors), the underlying mechanism remains unclear. Previously, in atrophic gastritis, a role of gastric emptying has been investigated, showing a delayed emptying both in the autoimmune^[28]^ and nonautoimmune atrophic gastritis.^[29]^ In addition, the reduced gastric acid secretion seems to be associated with the presence of dyspepsia^[30]^ and more recently, a Japanese study conducted on dyspeptic outpatients showed that hypochlorhydria was significantly associated with higher dyspeptic symptom scores and dysmotility-related scores, in particular, in female.^[31]^

In a systematic review evaluating the effects of proton pump inhibitors on gastric emptying, a consistent delaying effect of these drugs on gastric emptying of solid meals has been shown.^[32]^ Albeit the hypochlorhydria induced by proton pump inhibitors does not perfectly overlap the intragastric milieu of AG, these data seem to support the biological plausibility that the impaired gastric acid secretion might have a role in generating the dyspeptic symptoms, frequently present in AG.

Our results show that no relation between histological severity of oxyntic damage and the symptoms’ presence as shown in Fig. 1. However, we have to keep in mind that a close relationship between the degree of histological oxyntic damage and acid output has not been demonstrated, but the hypochlorhydria *per se*, as previously reported, should justify the occurrence of dyspeptic symptoms^[31]^ as reported in our study.

Dyspepsia is a chronic condition, which affects quality of life of patients, and it requires an early correct assessment in order to avoid unnecessary medical visits and repetition of unnecessary gastroscopy.^[33]^ Even if AG patients complain of GI symptoms, the clinical suspicion of AG is rarely raised on the basis of GI symptoms, even when dyspepsia is present. In our study, even if more than half of patients reported GI symptoms, less than a tenth underwent gastroscopy for GI symptoms.

Comparing patients with and without GI symptoms, the multivariate analysis shows that younger age, no smoking, and absence of anemia are factors associated to the presence of upper GI symptoms, particularly dyspepsia. On the contrary Soykan et al, ^[12]^ did not find any significant difference in terms of age, gender, autoimmune diseases, and presence of gastric autoantibodies when compare AG patients with or without symptoms.^[12]^ This might be important because clinical guidelines recommend gastroscopy with biopsies for dyspeptic patients over the age of 55 years and for patients with alarm symptoms (i.e., anemia),^[34]^ and younger dyspeptic patients without anemia generally are not referred to gastroscopy, thus possibly missing the diagnosis of AG.

We are aware of some limits of this study. The study was performed in an academic tertiary level center. This may have led to possible selection bias. The study population is representative of the population of Southern Europe, where the study has been performed. For these reasons, the results of this study possibly might not to be extended to the general population.

In conclusion, more than half of AG patients complained of GI symptoms, in particular, dyspepsia, alone in 70% or associated to GERD in 17.7% of symptomatic patients, confirming the significant occurrence of symptoms in this population. In autoimmune gastritis patients, the demonstrated association between early satiety and postprandial fullness with younger age, no smoker and not anemic status should be kept in mind in the work-up of these patients.

## Acknowledgment

The authors thank Maria Sofia Cattaruzza, Associate Professor of Epidemiology, for the statistical consultation.
